# Intestinal tuft cells regulate the ATM mediated DNA Damage response via Dclk1 dependent mechanism for crypt restitution following radiation injury

**DOI:** 10.1038/srep37667

**Published:** 2016-11-23

**Authors:** Parthasarathy Chandrakesan, Randal May, Nathaniel Weygant, Dongfeng Qu, William L. Berry, Sripathi M. Sureban, Naushad Ali, Chinthalapally Rao, Mark Huycke, Michael S. Bronze, Courtney W. Houchen

**Affiliations:** 1 Department of Medicine, University of Oklahoma Health Sciences Center, Oklahoma City, OK 73104, USA; 2 OU Cancer Institute, University of Oklahoma Health Sciences Center, Oklahoma City, OK 73104, USA; 3 Department of Veterans Affairs Medical Center, Oklahoma City, OK 73104, USA; 4 COARE Biotechnology, Inc., Oklahoma City, OK 73104, USA

## Abstract

Crypt epithelial survival and regeneration after injury require highly coordinated complex interplay between resident stem cells and diverse cell types. The function of Dclk1 expressing tuft cells regulating intestinal epithelial DNA damage response for cell survival/self-renewal after radiation-induced injury is unclear. Intestinal epithelial cells (IECs) were isolated and purified and utilized for experimental analysis. We found that small intestinal crypts of Villin^Cre^;Dclk1^f/f^ mice were hypoplastic and more apoptotic 24 h post-total body irradiation, a time when stem cell survival is p53-independent. Injury-induced ATM mediated DNA damage response, pro-survival genes, stem cell markers, and self-renewal ability for survival and restitution were reduced in the isolated intestinal epithelial cells. An even greater reduction in these signaling pathways was observed 3.5 days post-TBI, when peak crypt regeneration occurs. We found that interaction with Dclk1 is critical for ATM and COX2 activation in response to injury. We determined that Dclk1 expressing tuft cells regulate the whole intestinal epithelial cells following injury through paracrine mechanism. These findings suggest that intestinal tuft cells play an important role in regulating the ATM mediated DNA damage response, for epithelial cell survival/self-renewal via a Dclk1 dependent mechanism, and these processes are indispensable for restitution and function after severe radiation-induced injury.

In many mammalian gut tissues, the ability to regenerate an intact functional intestinal epithelium after severe mucosal injury requires the coordinated participation of surviving resident and reserve/rescue stem cells in connection with the non-epithelial and inflammatory cells within the crypt niche^1^,^2^,^3^. Intestinal stem cells (ISCs) maintain tissue homeostasis over the lifetime of the organism, and must respond to and recover from severe geno/cytotoxic insult^1^,^2^,^4^. Stem cells are thought to possess unique characteristics that may offer protection against acute and chronic injuries, promoting survival and, ultimately, repopulation of tissues^5^,^6^,^7^. This phenomenon has been readily observed in the gastrointestinal response to radiation injury^8^,^9^. Under normal conditions, these cells must self-renew to protect the genome of their more fully differentiated tissue progeny^4^,^10^. This process requires highly coordinated, complex interplay between resident tissue stem cells and the diverse cell types that reside and/or pass through the stem cell niche. The paracrine, autocrine, endocrine, and inflammatory signals that regulate this critical function are poorly understood. Furthermore, the regulatory mechanisms that govern the stem cell response at homeostasis and after injury are unknown.

We explored three fundamental questions: 1) how do intestinal epithelial cells (IECs)/ISCs respond to severe DNA damage? 2) do Dckl1-expressing tuft cells play a role in intestinal injury response? and 3) are there factors that reliably modify these responses? We used a radiation injury model to assess the functional IECs and ISCs response to high-dose (12 Gy) irradiation and built on our previous findings with Dclk1, a microtubule-associated kinase and tuft cell marker^11^,^12^,^13^,^14^. Dclk1 was originally thought to mark ISCs and gastric progenitors, but has more recently been demonstrated to mark tumor stem cells (TSCs) and label long-lived quiescent cells in the gut^15^,^16^. Under high-dose radiation and during DSS-induced colonic inflammation, lineage tracing could be observed in these cells^15^,^16^. These findings support the notion that these cells can act as stem cells under certain environmental conditions, even under non-neoplastic conditions.

Dclk1 marks tuft or brush cells, a 5th lineage in the small intestine^11^,^14^,^17^. Tuft cells are known to play a major role in taste discrimination and in response to noxious insults^18^. These cells have unique morphology and express Dclk1 and several additional unique proteins, including Cox-1, Cox-2, and trpm5^18^,^19^. Recent evidence suggests that tuft cells are chemosensory cells that capture locally transmitted microenvironmental signals that may regulate the secretory response governing cell fate during injury and, perhaps, homeostasis^20^,^21^. Very recently, we identified the molecular signature of Dclk1 expressing intestinal epithelial tuft cells, which display the hallmarks of quiescence and self-renewal^22^.

Although this function is speculative, our previous data evaluated the role of Dclk1 in tuft cells during the acute injury response. We demonstrated that intestinal deletion of Dclk1 does not delete tuft cells or confer a significant deleterious phenotype in adult mice, compared with their wild-type littermates^12^. None of these mice, however, survived longer than five days after TBI, due to an inability to restore epithelial barrier integrity^12^. Thus, we sought to more closely investigate the role of Dclk1 in crypt epithelial survival by regulating the DNA damage response (DDR), with an emphasis on evaluating crypt-specific tuft cells, with and without Dclk1 expression. Because, the best-known primary defense mechanism against the DNA damage associated with such exposures is the DNA damage response (DDR), which repairs the damaged DNA and increased the survival of epithelial cells.

The DDR of mouse ISCs involves the tumor suppressor protein p53 within the first 6 h after irradiation. However, by 24 h after irradiation, stem cell survival is p53-independent^23^. This time point is likely the last chance for surviving stem cells to participate in epithelial restitution of the gut and survival of the animal, if the appropriate immune-supportive features can be restored^24^. There is some evidence that ISCs are resistant to radiation-induced apoptosis^25^,^26^. DDR is primarily mediated by phosphatidylinositol-3-kinase-like protein kinase (PIKKs) family members, ataxia-telangiectasia mutated (ATM) and ataxia telangiectasia and Rad3-related protein (ATR)^27^. ATM pathway for homologous recombination (HR) repair is activated following irradiation and, likely, injury. ATR pathway for non-homologous end-joining (NHEJ) is considered less accurate and more error-prone than HR^28^. However, recent studies confirmed that HR is the predominant DSB repair pathway in hESCs and mESCs, in contrast to those in differentiated cells^9^,^29^,^30^. Furthermore, stem cells must maintain their genomic integrity to preserve appropriate self-renewal under homeostasis and injury^7^.

These findings led us to similar investigations in the mouse gut. The obvious potential adverse effect of DDR on DSBs in IECs and ISCs are critical for recovering from radiation-induced injury for appropriate survival and self-renewal. In the current study, we exposed Villin^Cre^;Dclk1^f/f^ mice and Dclk1^f/f^ littermates to lethal dose 12 Gy TBI. Twenty-four hours and eighty four hours post-TBI, IECs were isolated without stromal contamination and utilized for protein and gene expression analysis along with functional analysis to identify the effect of Dclk1 loss in tuft cells on intestinal epithelial cell survival, pluripotency, and DDR. We also evaluated the self-renewal of IECs, using enteroid growth as a measure of whether IECs isolated from Villin^Cre^;Dclk1^f/f^ mice have increased sensitivity to radiation-induced loss of stemness. Further, we utilized *in vitro* cell culture studies to identify the importance of Dclk1 expressing in YAMC cells regulating DDR and cell survival. Our observations revealed that Dclk1 expression in tuft cells enhance intestinal epithelial radio-resistance and self-renewal/survival through paracrine regulation and activation of ATM mediated DDR.

## Results

### Intestinal epithelial-specific Dclk1 knockout exhibits intestinal crypt hypoplasia and impaired expression of putative stem cell markers 24 h post-TBI

**Figure 1:**
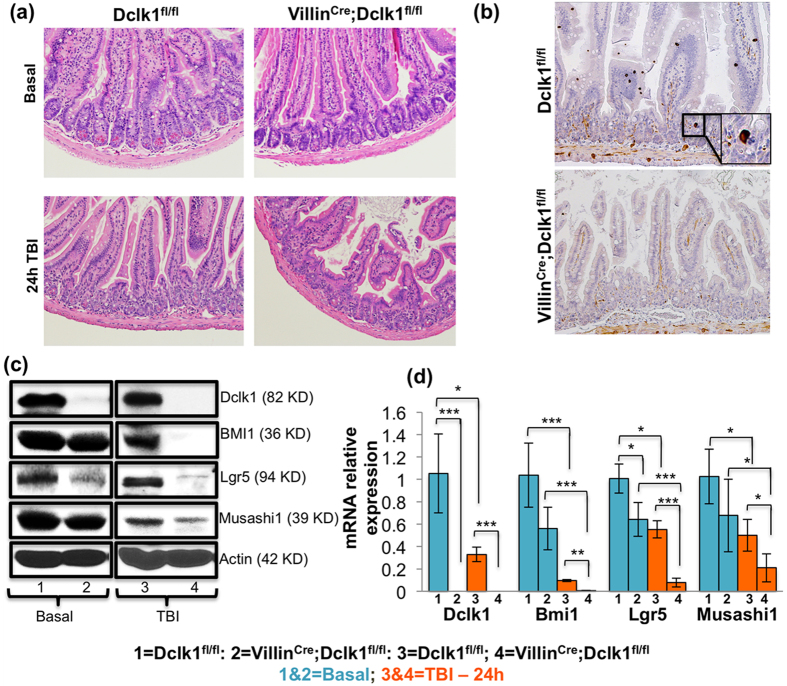
Intestine-specific Dclk1-KO mice exhibit intestinal crypt epithelial hypoplasia and impaired stem cell population 24 h post-TBI. (**a,b**) Intestinal tissue sections from Villin^Cre^;Dclk1^f/f^ mice and Dclk1^f/f^ mice, before and after TBI (24 h), were stained for H&E and anti-Dclk1. (**c**) Western blot analysis of protein expression of stem cell markers Bmi1, Lgr5, Msi1, and Dclk1 in isolated IECs from Villin^Cre^;Dclk1^f/f^ and Dclk1^f/f^ mice, before and 24 h after TBI. (**d**) RT-PCR analysis of mRNA expression of stem cell markers Bmi1, Lgr5, Msi1, and Dclk1 in isolated IECs from Villin^Cre^;Dclk1^f/f^ mice and Dclk1^f/f^ mice, before and 24 h after TBI. All quantitative data are expressed as means ± *SD* of a minimum of three independent experiments. *P* values of< 0.05 = *, <0.01 = ** and 0.001 = *** were considered statistically significant.

Dclk1 expression is restricted to tuft cells in the small intestine. To explore the possible functional role of Dclk1 expression in tuft cells regulating the ISC response with respect to crypt epithelial proliferation and differentiation, we subjected Villin^Cre^;Dclk1^f/f^ mice, a model that lacks Dclk1 expression in the whole intestinal epithelial cells (Fig. 1b), and Dclk1^f/f^ mice (wild-type littermates) to 12 Gy lethal TBI to induce severe epithelial injury. In the absence of injury, crypt architecture and intestinal histology were not notably different in Villin^Cre^;Dclk1^f/f^ mice compared with Dclk1^f/f^ mice (Fig. 1a), indicating that Dclk1-expressing tuft cells may be dispensable for crypt homeostasis under normal conditions. We compared the crypt architecture and intestinal histology and DNA damage between the Villin^Cre^ mice and Dclk1^f/f^ mice and found no change in phenotype between these mice (Suppl. Fig. 1). At 24 h post-TBI, intestinal crypt architecture and epithelial arrangement in the crypt were deranged, and a reduction in crypt epithelial cell number was observed in Dclk1^f/f^ mice (Fig. 1a)^31^. Villin^Cre^;Dclk1^f/f^ mice displayed acute crypt hypoplasia, markedly distorted and shortened crypts that become widely separated from the underlying muscularis mucosae, and a dramatic reduction in IECs, indicating that Dclk1 expression is critical in maintaining crypt epithelial morphology and cell number 24 h after severe genotoxic injury (Fig. 1a). The expression of ISC markers Lgr5, Bmi1, and Musashi1 was also markedly reduced in the IECs of Villin^Cre^;Dclk1^f/f^ mice compared with Dclk1^f/f^ mice 24 h post-TBI (Fig. 1c,d), suggesting that Lgr5^+^, Bmi1^+^, and Musashi1^+^ stem cells were reduced in the Villin^Cre^;Dclk1^f/f^ animals. Lgr5 expression was reduced even before TBI, suggesting that the regulation of Lgr5 expression and or Lgr5^+^ stem cells under homeostatic conditions may require Dclk1 expression in tuft cells in the intestine (Fig. 1c,d). These data suggest that Dclk1 is required in tuft cells to protect and/or maintain crypt epithelial morphology after injury, perhaps it also suggested that possible coordination between Dclk1 expressing tuft cells and additional stem cell proteins or stem cells is prerequisite.

### Deficiency in the stemness of Villin^Cre^;Dclk1^f/f^ mice after TBI-induced injury

**Figure 2:**
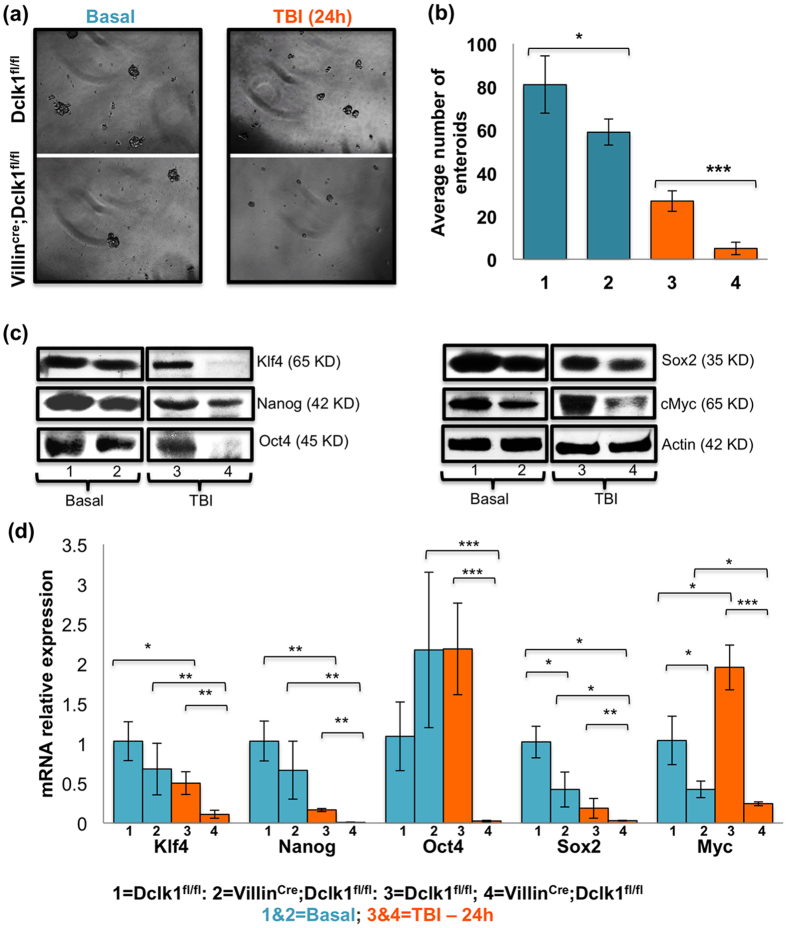
Dclk1 expressing tuft cells are required to enhance crypt epithelial stemness 24 h post-TBI. (**a**) Enteroid formation assay: IECs isolated from Villin^Cre^;Dclk1^f/f^ and Dclk1^f/f^ mice, before and 24 h after TBI were directly embedded in 0.3% soft agar. (**b**) Bar graph represents the average number of enteroids formed from IECs isolated from Villin^Cre^;Dclk1^f/f^ and Dclk1^f/f^ mice, before and 24 h after TBI. (**c**) Western blot analysis of protein expression of pluripotency factors Klf4, Nanog, Oct4, Sox2, and cMyc in IECs isolated from Villin^Cre^;Dclk1^f/f^ and Dclk1^f/f^ mice, before and 24 h after TBI. (**d**) RT-PCR analysis of mRNA expression of pluripotency factors Klf4, Nanog, Oct4, Sox2, and cMyc in IECs isolated from Villin^Cre^;Dclk1^f/f^ and Dclk1^f/f^ mice, before and 24 h after TBI. All quantitative data are expressed as means ± *SD* of a minimum of three independent experiments. *P* values of <0.05 = * and < 0.01 = **, 0.001 = *** were considered statistically significant.

Pluripotency is a central, well-defined feature of stem cells. Analysis of the pluripotency factors expressed in IECs and their self-renewal ability during the injury response may reveal the coordinated molecular pathways that regulate injury/wound repair^32^,^33^. To understand how Dclk1 expression in the tuft cells contribute to IECs self-renewal and pluripotency at 24 h post-TBI, we investigated the enterosphere-forming ability and expression of pluripotency factors. IECs isolated from Villin^Cre^;Dclk1^f/f^ mice formed 5-fold fewer enterospheres than Dclk1^f/f^ mice (*p* < 0.0001; Fig. 2a,b). Before TBI, significantly fewer enterospheres were formed in Villin^Cre^;Dclk1^f/f^ mice than in Dclk1^f/f^ mice (*p* < 0.01; Fig. 2a,b). Thus, the loss of Dclk1 expression in tuft cells reduced the clonogenic capacity of resident crypt epithelial cells, suggesting the paracrine function of tuft cells prerequisite Dclk1 expression to maintain the self-renewal ability of IECs. Before TBI, expression of pluripotency factors Sox2 and Myc were significantly lower in IECs from Villin^Cre^;Dclk1^f/f^ mice than Dclk1^f/f^ mice (*p* < 0.001; Fig. 2c,d). This finding may represent an additional mechanism of reduced self-renewal during homeostasis. However, 24 h post-TBI, the mRNA and protein levels of Nanog and Sox2 (*p* < 0.001), and Oct4, Klf4, and Myc (*p* < 0.0001) were significantly downregulated in IECs from Villin^Cre^;Dclk1^f/f^ mice compared with Dclk1^f/f^ mice (Fig. 2c,d). These results suggest that Dclk1 expression in tuft cells may be required for the paracrine maintenance of intestinal epithelial pluripotency and/or the regeneration of sufficient pluripotent cells to participate in epithelial restitution. However, the secretory factors responsible for the paracrine function of Dclk1 expressing tuft cells need further investigation.

Following severe genotoxic or cytotoxic injuries, ISC function is regulated by enhanced expression of pro-survival factors and activation of key signaling pathways that promote rapid growth and differentiation based on signals emanating from or around the crypt niche microenvironment^34^,^35^. Akt/mTOR expression, critical for cell survival, efficient cellular energy metabolism and pluripotency, were downregulated in IECs from Villin^Cre^;Dclk1^f/f^ mice 24 h post-TBI (Suppl. Fig. 2). Like Akt/mTOR, the Notch, beta-catenin, and RelA signaling pathways, their downstream targets Hes1, Tcf4, CXCL1, and CyclindD1, were significantly downregulated in IECs from Villin^Cre^;Dclk1^f/f^ mice compared with Dclk1^f/f^ mice, 24 h post-TBI (Suppl. Figs 3 and 4). These pathways are critical for normal cell function, survival, and self-renewal^32^,^33^. These pro-survival signals not only display autocrine function, but also play a paracrine role in protecting cell survival, particularly in response to injury^36^,^37^,^38^,^39^.

**Figure 3:**
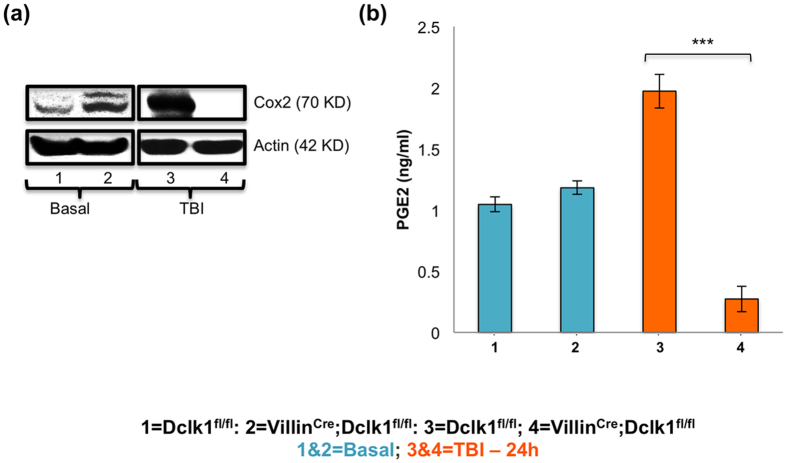
COX2 signaling requires Dclk1 expression in the intestinal epithelial tuft cells after injury. (**a**) Western blot analysis of protein expression of Cox2 in the isolated IECs from Villin^Cre^;Dclk1^f/f^ and Dclk1^f/f^ mice, before and 24 h after TBI. (**b**) Serum level of PGE2 from Villin^Cre^;Dclk1^f/f^ and Dclk1^f/f^ mice, before and 24 h after TBI. All quantitative data are expressed as means ± *SD* of a minimum of three independent experiments. *P* values of <0.05 = *, <0.01 = ** and 0.001 = *** were considered statistically significant.

Recently, it was reported that COX1/2 is predominantly expressed in the tuft cells of the small intestine^18^. COX2 signaling and prostaglandin secretions are important for the survival of IECs and ISCs after injury^40^,^41^. In the present study, we found decreased expression of COX2 in the IECs of Villin^Cre^;Dclk1^f/f^ mice compared with Dclk1^f/f^ mice, 24 h post-TBI (Fig. 3a). Further, under homeostasis, COX2 expression was increased in the IECs of Villin^Cre^;Dclk1^f/f^ mice compared with Dclk1^f/f^ mice, suggesting either an adaptive response to support the tuft cells of Dclk1 loss and/or unregulated expression due to lack of Dclk1 inhibition (Fig. 3a), which need further investigation. Serum levels of PGE2 were decreased in the Villin^Cre^;Dclk1^f/f^ mice compared with Dclk1^f/f^ mice, 24 h post-TBI (Fig. 3b). These findings suggest that Dclk1 expression in tuft cells may regulate the PGE2 levels in the intestine via COX2 signaling in response to injury as a paracrine mechanism.

### Defects in intestinal epithelial barrier function in Villin^Cre^;Dclk1^f/f^ mice 24 h after TBI-induced injury

**Figure 4:**
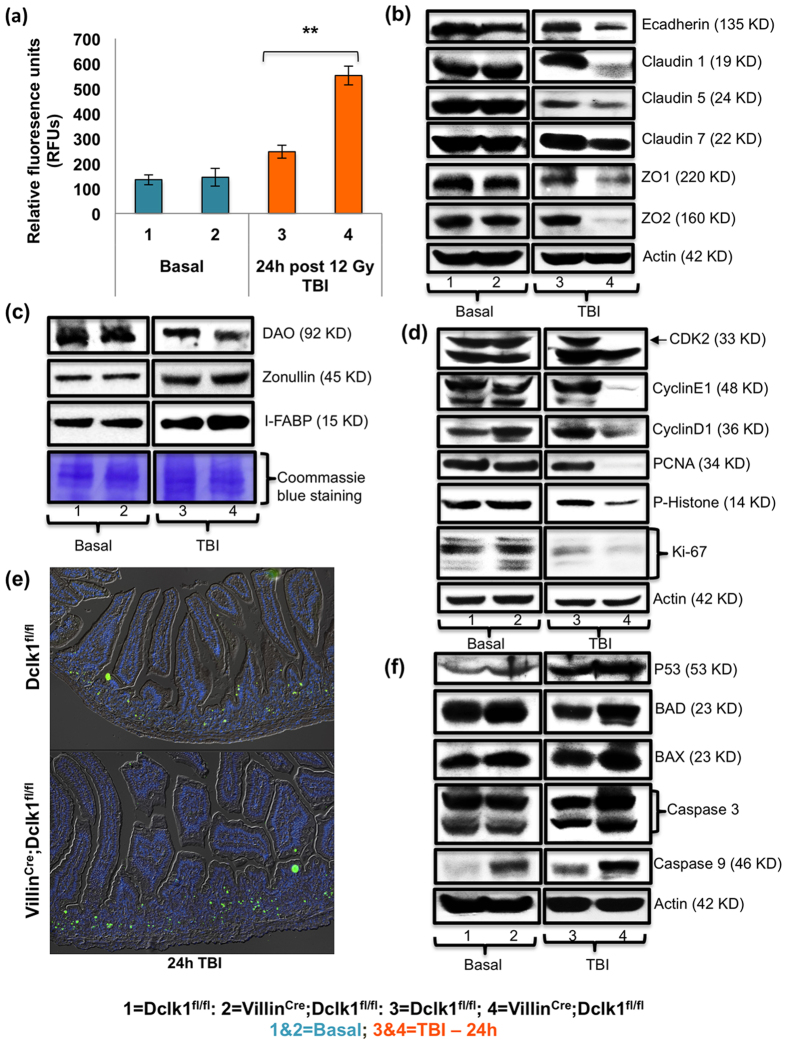
Dclk1 deficiency in tuft cells results in defective epithelial barrier and survival 24 h post-TBI. (**a**) At baseline and 24 h post-TBI Villin^Cre^;Dclk1^f/f^ and Dclk1^f/f^ mice were gavaged with FITC-labeled dextran (dose = 80 mg/100 g of body weight) 4 hours prior to being killed. Blood was collected *via* cardiac puncture at the time of death to examine FITC dextran uptake. (**b**) Western blot analysis of protein expression of adherens junction protein e-cadherin and tight junction proteins Claudin 1, 5 and 7, and Zona Occludin 1 and 2 in IECs isolated from Villin^Cre^;Dclk1^f/f^ and Dclk1^f/f^ mice, before and 24 h after TBI. (**c**) Western blot analysis of serum biomarkers zonulin, diamine oxidase (DAO) and IFABP from Villin^Cre^;Dclk1^f/f^ and Dclk1^f/f^ mice, before and 24 h after TBI. (**d,f**) To assess IEC cycling status and apoptosis, western blot analysis was performed to identify the protein expression levels of Cdk2, CyclinE1, CyclinD1, PCNA, phospho histone, ki-67, p53, Bad, Bax, and Caspase 3 and 9 in IECs isolated from Villin^Cre^;Dclk1^f/f^ and Dclk1^f/f^ mice, before and 24 h after TBI. (**e**) Intestinal tissue sections from Villin^Cre^;Dclk1^f/f^ mice and Dclk1^f/f^ mice, before and 24 h after TBI, were TUNEL-stained to analyze IEC apoptosis. All quantitative data are expressed as means ± *SD* of a minimum of three independent experiments. *P* values of <0.05 = *, <0.01 = **, and 0.001 = *** were considered statistically significant.

To determine the physiologic mechanism by which Dclk1 maintains the epithelial barrier function critical for epithelial cell survival and restitution, we compared the expression patterns of key junctional proteins and correlated these patterns with barrier function using the FITC assay protocol, before and 24 h after TBI, in Villin^Cre^;Dclk1^f/f^ and Dclk1^f/f^ mice. We observed a marked increase (*p* < 0.0001) in the intestinal permeability of Villin^Cre^;Dclk1^f/f^ mice compared with Dclk1^f/f^ mice 24 h post-TBI (Fig. 4a). The increased intestinal permeability due to loss of Dclk1 suggests that Dclk1 expression in tuft cells during injury is the vital factor required for functional intestinal barrier. We investigated the response of intestinal junctional proteins to epithelial damage resulting from TBI. Tight junction proteins claudin-1 and ZO-2 were decreased ~10 fold, and claudin-5, −7, and ZO-1 were decreased ~2 fold in IECs from Villin^Cre^;Dclk1^f/f^ mice compared with Dclk1^f/f^ mice, 24 h post-TBI (Fig. 4b). The adherens junction protein E-cadherin was significantly reduced (~3 fold) in Villin^Cre^;Dclk1^f/f^ mice compared with Dclk1^f/f^ mice, 24 h post-TBI (Fig. 4b). Furthermore, serum biomarkers of intestinal permeability, including zonulin, diamine oxidase (DAO), and intestinal fatty acid binding protein (IFABP), were significantly altered in the serum collected from Villin^Cre^;Dclk1^f/f^ mice compared with Dclk1^f/f^ mice, 24 h post-TBI (Fig. 4c). However, under homeostasis, these junctional proteins and the intestinal permeability were not significantly altered in Villin^Cre^;Dclk1^f/f^ mice, suggesting that Dclk1 expression in tuft cells are likely required for its function during injury to maintain the integrity of the intestinal barrier and to support radio-resistance for epithelial replenishment, mucosal restitution, and recovery.

### Increased apoptosis and decreased cell proliferation in Villin^Cre^;Dclk1^f/f^ mice post-TBI

It is recently suggested that high-dose radiation causes loss of cycling stem cells and activates resistant quiescent stem cells^8^. Crypt Dclk1^+^ tuft cells are relatively resistant to low-dose < 8 Gy radiation^17^. Here, we investigated the importance of Dclk1-expressing tuft cells in regulating IEC cycling for replenishment after 12 Gy TBI. Cell cycle regulators cdk2, cyclin E1, and cyclin D1 were ~5–6 fold lower in IECs from Villin^Cre^;Dclk1^f/f^ mice than Dclk1^f/f^ mice, 24 h post-TBI (Fig. 4d). Expression of cell proliferation markers PCNA, phospho-histone, and ki-67 showed a marked reduction in Villin^Cre^;Dclk1^f/f^ IECs compared with Dclk1^f/f^ mice, 24 h post-TBI (Fig. 4d). Thus, the loss of Dclk1 expression in tuft cells reduced intestinal epithelial proliferation after TBI by altering the expression of cell cycle regulators. However, under homeostasis, IEC proliferation, determined by phospho-histone, ki-67, and cyclin D1 expression, was higher in Villin^Cre^;Dclk1^f/f^ mice than in Dclk1^f/f^ mice (Fig. 4d). These data suggest that Dclk1 in tuft cells may be involved in maintaining cell quiescence and proliferation by modulating cell cycle regulators. Nevertheless, the release from quiescence after injury was severely impaired after loss of Dclk1.

Next, we tested whether the crypt hypoplasia and decreased cell proliferation after epithelial injury were associated with changes in apoptosis. Increased terminal deoxynucleotidyl transferase-mediated deoxyuridine-triphosphate nick end labeling (TUNEL) staining in the intestine (~4–5 TUNEL-positive cells per crypt; Fig. 4e, Suppl. Fig. 5) and expression and activity of caspase 3 and 9 in IECs indicated severe apoptosis in Villin^Cre^;Dclk1^f/f^ mice compared with Dclk1^f/f^ mice, 24 h post-TBI (Fig. 4f, Suppl. Fig. 6). We observed a marginal increase (~0.5–1 fold) in p53 expression in IECs from Villin^Cre^;Dclk1^f/f^ mice compared with Dclk1^f/f^ mice post-TBI. However, expression of p53-regulated targets Bax and Bad were significantly increased (~2–3 fold) in IECs from Villin^Cre^;Dclk1^f/f^ mice (Fig. 4f). The loss of Dclk1 expression in tuft cells during TBI-induced epithelial injury results in massive deregulation in the coordination of cell cycle arrest mechanisms and p53-independent apoptosis.

### Dclk1 regulates DNA damage response (DDR) and maintains genomic integrity in IECs

**Figure 5:**
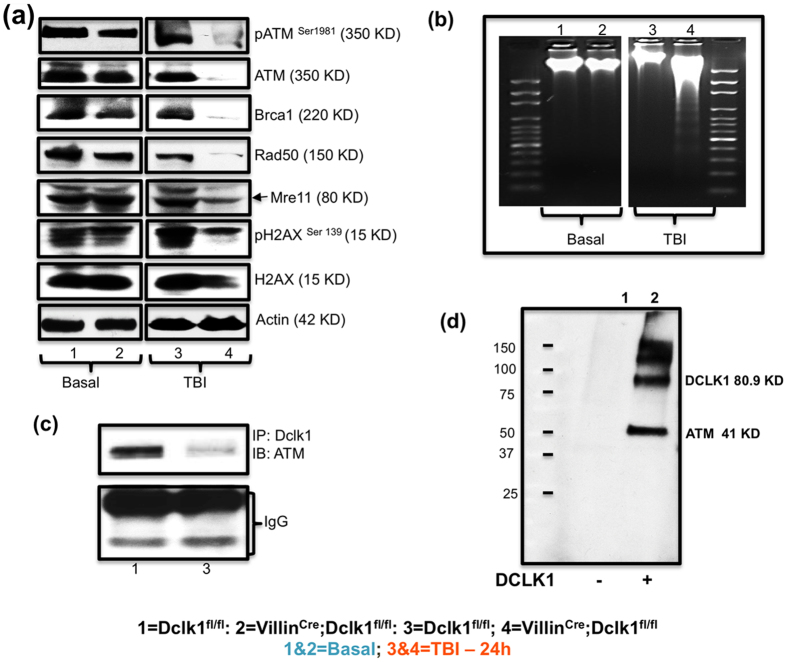
Dclk1 regulates DDR by activating the ATM pathway, and maintains IECs DNA integrity 24 h post-TBI. (**a**) Western blot analysis was performed to identify the protein expression levels of phospho ATM (ser 1981), total ATM, Brca1, Rad50, Mre11, gammaH2AX, and total H2AX in IECs isolated from Villin^Cre^;Dclk1^f/f^ and Dclk1^f/f^ mice, before and 24 h after TBI. (**b)** Intestinal tissues from Villin^Cre^;Dclk1^f/f^ mice and Dclk1^f/f^, mice before and 24 h after TBI, were used to isolate DNA for the agarose gel DNA fragmentation assay. (**c**) Co-immunoprecipitation: IEC protein extracts from Dclk1^f/f^ mice at homeostasis and after TBI were co-immunoprecipitated with anti-Dclk1 and blotted with antibody to ATM. Lower bands represent IgG heavy and short chains. (**d**) Photo-cross-linking label transfer assays: Photo-activation and transfer of biotin tagged label from the bait DCLK1 to ATM, which was identified probing the samples with streptavidin HRP conjugate. Confirmatory analysis identifies the interaction between Dclk1 and ATM. All quantitative data are expressed as means ± *SD* of a minimum of three independent experiments. *P* values of <0.05 = *, <0.01 = **, and 0.001 = *** were considered statistically significant.

Deficits in DNA damage response enhance intestinal radiosensitivity. However, stem cells and tuft cells show more resistance to injury-induced DNA damage^4^,^13^,^16^. Their functional demand and longevity are associated with effective DDR to ensure genomic integrity^7^,^42^. To address this, we investigated the activation of HR, the predominant double-strand breaks (DSBs) repair pathway for radiation-induced DSBs^7^. Expression of ATM, gamma-H2AX, and the adopter proteins BRCA1, Rad50, and MRE11 was reduced in IECs from Villin^Cre^;Dclk1^f/f^ mice compared with Dclk1^f/f^ mice, 24 h post-TBI (Fig. 5a). These data suggest that ATM mediated DDR protects against TBI-induced IEC and ISC loss, while Dclk1 expression in tuft cells may play a vital coordination role in regulating intestinal epithelial DDR. Dclk1 expression appears to develop radioresistant and anti-apoptotic functions may be through paracrine mechanisms that maintain DNA integrity after TBI-induced epithelial injury, a finding that is clearly supported by the results of our DNA fragmentation assay (Fig. 5b).

Prostaglandins (PGs) are synthesized from arachidonic acid either by cyclooxygenase-1 (COX-1) or cyclooxygenase-2 (COX-2). PGE2 *via* prostaglandin E2 receptor (EP2) displays strong radio-protective effects on the intestinal epithelium^43^. Thus, loss of COX2 signaling after TBI in the IECs of Villin^Cre^;Dclk1^f/f^ mice and reduced serum PGE2 suggests that Dclk1 is critical to enhance COX2 signaling and PGE2 expression in response to injury to protect cell damage and their genomic integrity (Fig. 3a,b).

We observed reduced ATM phosphorylation in IECs from Villin^Cre^;Dclk1^f/f^ mice under homeostasis (Fig. 5a). To better determine the direct role of Dclk1 on ATM activation, we performed a co-immunoprecipitation (CO-IP) assay. We hypothesized that Dclk1, a kinase protein, might interact with ATM, as its phosphorylation leads to activation. CO-IP revealed protein-protein interaction in IECs from Dclk1^f/f^ mice, before and 24 h post-TBI, confirming that Dclk1 and ATM interact, and demonstrating that this interaction was markedly reduced 24 h post-TBI (Fig. 5c). To further confirm the novel finding of Dclk1-ATM interaction, we performed label transfer protein interaction analysis utilizing purified Dclk1, and found that the label is transferred to ATM after interaction (Fig. 5d). These findings strongly support that there is a direct link between Dclk1 expression and enhancement of DDR by activating the ATM and its downstream targets to protect IECs and ISCs.

### Villin^Cre^;Dclk1^f/f^ mice exhibit impaired intestinal epithelial renewal, epithelial permeability, and survival 84 h post-TBI

Twenty-four hours post-TBI, the IECs from Villin^Cre^;Dclk1^f/f^ mice showed dysregulated survival signaling and defective self-renewal, leading to crypt hypoplasia and increased apoptosis. This is the critical time point when p53-independent survival of ISCs is required for normal recovery and restoration of intact crypt/villus morphology in the regenerative intestine. The distinctive tissue response to irradiation in the intestine involves apoptosis of IECs and ISCs, resulting in crypt depletion on day 1, followed by regeneration of surviving crypt clonogens, producing crypt microcolony formation at 3–4 days^4^,^44^. We examined the extent of the impact of Dclk1 loss in tuft cells 3.5 days (84 h) post-TBI. This line of investigation will provide us with further understanding of the importance of Dclk1expressing tuft cells in coordinated regulation of IEC self-renewal and survival for restitution.

**Figure 6:**
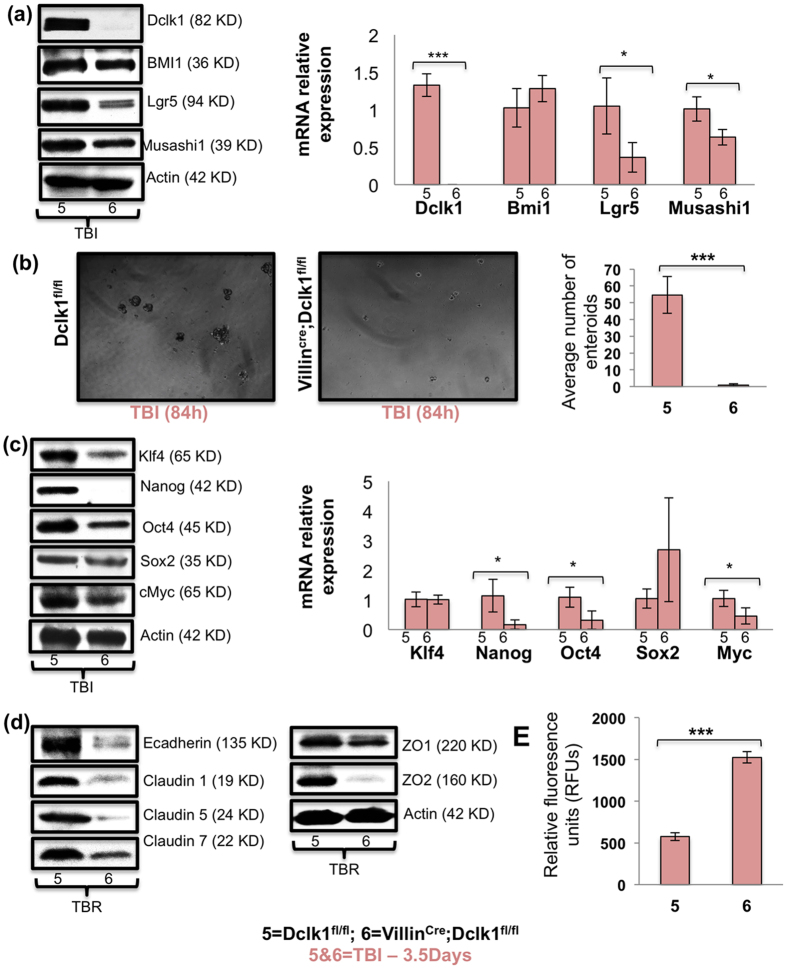
Dclk1 KO mice exhibit impaired intestinal epithelial self-renewal and epithelial barrier function 84 h post-TBI. (**a**) Western blot analysis of protein expression of stem cell markers Bmi1, Lgr5, Msi1, and Dclk1, in IECs isolated from Villin^Cre^;Dclk1^f/f^ and Dclk1^f/f^ mice 84 h post-TBI. RT-PCR analysis of mRNA expression of stem cell markers Bmi1, Lgr5, Msi1, and Dclk1, in IECs isolated from Villin^Cre^;Dclk1^f/f^ and Dclk1^f/f^ mice 84 h post-TBI. (**b**) Enteroid formation assay: IECs isolated from Villin^Cre^;Dclk1^f/f^ and Dclk1^f/f^ mice 84 h after TBI were directly embedded in 0.3% soft agar. Bar graph represents the average number of enteroids formed from IECs isolated from Villin^Cre^;Dclk1^f/f^ and Dclk1^f/f^ mice 84 h post-TBI. (**c**) Western blot analysis of protein expression of pluripotency factors Klf4, Nanog, Oct4, Sox2, and cMyc in IECs isolated from Villin^Cre^;Dclk1^f/f^ and Dclk1^f/f^ mice 84 h post-TBI. RT-PCR analysis of mRNA expression of pluripotency factors Klf4, Nanog, Oct4, Sox2, and cMyc in IECs isolated from Villin^Cre^;Dclk1^f/f^ and Dclk1^f/f^ mice 84 h post-TBI. (**d**) Western blot analysis of protein expression of e-cadherin, Claudin 1, 5 and 7, and Zona Occludin 1 and 2 in IECs isolated from Villin^Cre^;Dclk1^f/f^ and Dclk1^f/f^ mice 84 h post-TBI. (**e**) Fluorometric analysis of cardiac blood collected 4 hours after FITC gavage in Villin^Cre^;Dclk1^f/f^ and Dclk1^f/f^ mice 84 h post-TBI. All quantitative data are expressed as means ± *SD* of a minimum of three independent experiments. *P* values of <0.05 = *, < 0.01 = **, and 0.001 = *** were considered statistically significant.

**Figure 7:**
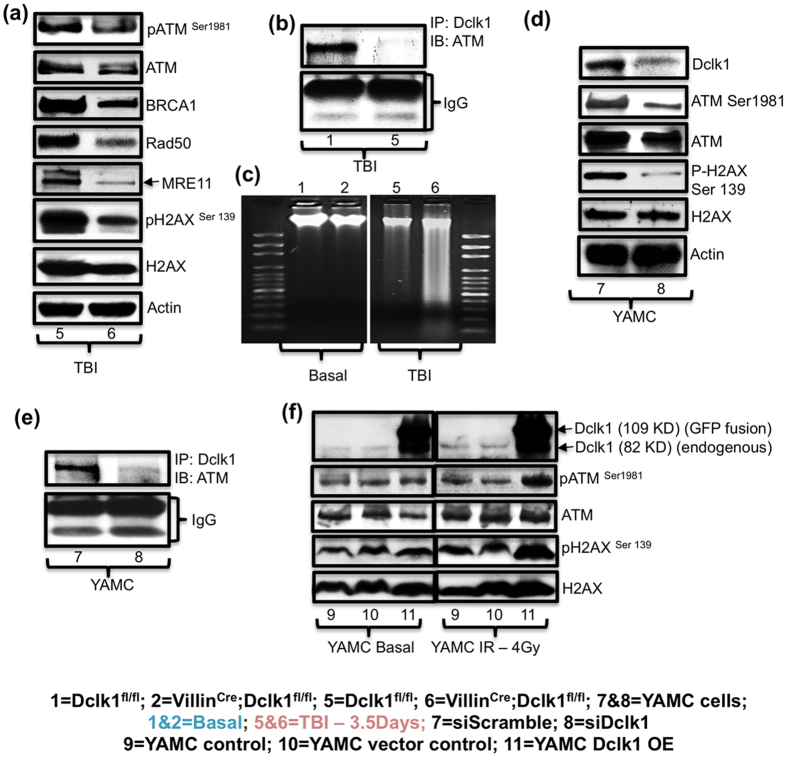
Dclk1 regulates ATM mediated intestinal epithelial DDR 84 h post-TBI. (**a**) Western blot analysis was performed to identify the protein expression level of phospho ATM (ser 1981), total ATM, Brca1, Rad50, Mre11, gammaH2AX, and total H2AX in IECs isolated from Villin^Cre^;Dclk1^f/f^ and Dclk1^f/f^ mice 84 h post-TBI. **(b**) Co-immunoprecipitation: IEC protein extracts from Dclk1^f/f^ mice at homeostasis and 84 h post-TBI were co-immunoprecipitated with anti-Dclk1 and blotted with antibody to ATM. Lower bands represent IgG heavy and short chains. (**c**) Intestinal tissues from Villin^Cre^;Dclk1^f/f^ mice and Dclk1^f/f^ mice, before and 84 h after TBI, were used to isolate DNA for an agarose gel DNA fragmentation assay. (**d**) Western blot analysis was performed to identify the DNA DSB repair signaling in YAMC cells 48 h after siDclk1 treatment *in vitro*. (**e**) Co-immunoprecipitation: Protein extracts from YAMC cells after siDclk1 and siScramble treatment were co-immunoprecipitated with anti-Dclk1 and blotted with antibody to ATM. Lower bands represent IgG heavy and short chains. **(f)** The DNA DSB repair response to radiation (4 Gy) injury and at basal conditions was assessed in Dclk1-overexpressing YAMC cells, before and 48 h post-radiation exposure *in vitro*.

The expression of ISC markers was greatly reduced in the intestines of Villin^Cre^;Dclk1^f/f^ mice compared with Dclk1^f/f^ mice, even 84 h post-TBI (Fig. 6a). IEC self-renewal was reduced to undetermined levels in Villin^Cre^;Dclk1^f/f^ mice compared with Dclk1^f/f^ mice, as shown by clonogenic assay (Fig. 6b). Expression of intestinal pluripotency factors was greatly downregulated in Villin^Cre^;Dclk1^f/f^ mice 84 h post-TBI (Fig. 6c). Furthermore, intestinal epithelial survival signaling pathways, including the Akt/mTOR, Notch, beta-catenin, RelA signaling pathways, and Hes1, Tcf4, CXCL1, CyclindD1, COX2 expression were dramatically reduced in the IECs of Villin^Cre^;Dclk1^f/f^ mice 84 h post-TBI (Suppl. Fig. 7). Compared with Dclk1^f/f^ mice, there was a substantial increase (*p* < 0.0001) in intestinal permeability (FITC-assay), representing the loss of barrier function, in Villin^Cre^;Dclk1^f/f^ mice 84 h post-TBI (Fig. 6d). Levels of tight junction proteins claudin-1, 5, 7, and ZO-2 were ~5–8 fold lower in IECs from Villin^Cre^;Dclk1^f/f^ mice than in Dclk1^f/f^ mice (Fig. 6e). We also observed massive deficiencies of gamma-H2AX and phospho-ATM and its downstream signaling molecules in IECs from Villin^Cre^;Dclk1^f/f^ mice (Fig. 7a). CO-IP data at 84 h post-TBI revealed that Dclk1-ATM interaction was still lower than that found in Dclk1^f/f^ mice at baseline (Fig. 7b). Results from a DNA fragmentation assay (Fig. 7c) revealed that Dclk1 is critical for radioresistance, maintaining DNA integrity even 84 h post-TBI. From the results of CO-IP conducted 24 h and 84 h post-TBI, we theorize that Dclk1 may have a direct role in the activation of ATM and its downstream targets for cell survival, and tuft cell survival in particular, for longevity and radioprotection after radiation injury.

To further confirm that loss of Dclk1 leads to reduced ATM activation and/or phosphorylation, we knocked down Dclk1 in YAMC cells and performed CO-IP. ATM phosphorylation was strongly associated with Dclk1 protein level (Fig. 7d,e). Furthermore, we determined that phospho-ATM and gamma-H2AX were reduced after Dclk1 knockdown (Fig. 7d). However, Dclk1 overexpression in YAMC cells enhanced the DDR 48 h post-radiation. We also observed increased expression of phospho-ATM and gamma-H2AX in Dclk1-overexpressing YAMC cells 48 h post-radiation (Fig. 7f). However, no change in ATM and H2AX expression levels was observed in either control or Dclk1-overexpressing cells at baseline (Fig. 7f). This finding suggests that the interaction of Dclk1 and ATM increased after injury.

### Dclk1 regulates ATM mediated DDR in response to irradiation injury *in vitro*

**Figure 8:**
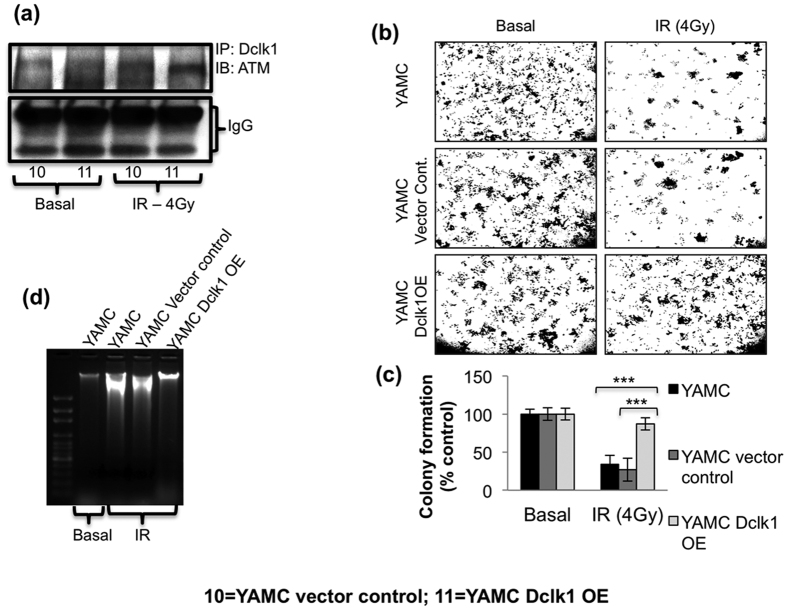
ATM activation depends on Dclk1 expression and interaction to maintain DNA integrity and cell survival. (**a**) Co-immunoprecipitation: Protein extracts from vector control YAMC cells and Dclk1-overexpressing cells at basal conditions and 48 h post-radiation (4 Gy) were co-immunoprecipitated with anti-Dclk1 and blotted with antibody to ATM. Lower bands represent IgG heavy and short chains. (**b**) Colony formation assay: We assessed the colony-forming ability of control, vector control, and Dclk1-overexpressing YAMC cells at baseline and 48 h post-radiation (4 Gy). (**c**) Bar graph represents the percent colony formation of control, vector control, and Dclk1-overexpressing YAMC cells 48 h post-radiation, compared with these cells at baseline. (**d**) DNA was isolated from YAMC cells of representative groups for an agarose gel DNA fragmentation assay. All quantitative data are expressed as means ± *SD* of a minimum of three independent experiments. *P* values of <0.05 = *, <0.01 = **, and 0.001 = *** were considered statistically significant.

To test whether the ATM phosphorylation level is directly associated with Dclk1 levels after injury, Dclk1-overexpressing YAMC cells and vector control cells were exposed to radiation and utilized for CO-IP (Fig. 8a). The interaction of ATM and Dclk1 was significantly enhanced after radiation-induced injury. CO-IP revealed that ATM phosphorylation might be directly associated with Dclk1 expression levels after injury. To further test the involvement of Dclk1 in DDR for cell radioprotection and survival/viability, we conducted a colony formation assay post irradiation injury, and found that Dclk1-overexpressing YAMC cells form the same number of colonies as do complete control YAMC cells (not exposed to irradiation), demonstrating that Dclk1-overexpressing cells display enhanced radioprotection after radiation injury (Fig. 8b,c). To examine whether the Dclk1 level is distinct for DNA integrity after injury, Dclk1-overexpressing YAMC cells and vector control cells were exposed to radiation and utilized for a DNA fragmentation assay (Fig. 8d). Compared with vector control cells exposed to irradiation, Dclk1-overexpressing YAMC cells had less DNA damage.

### Dclk1 regulates COX2 signaling and PGE2 expression in response to irradiation injury *in vitro*

**Figure 9:**
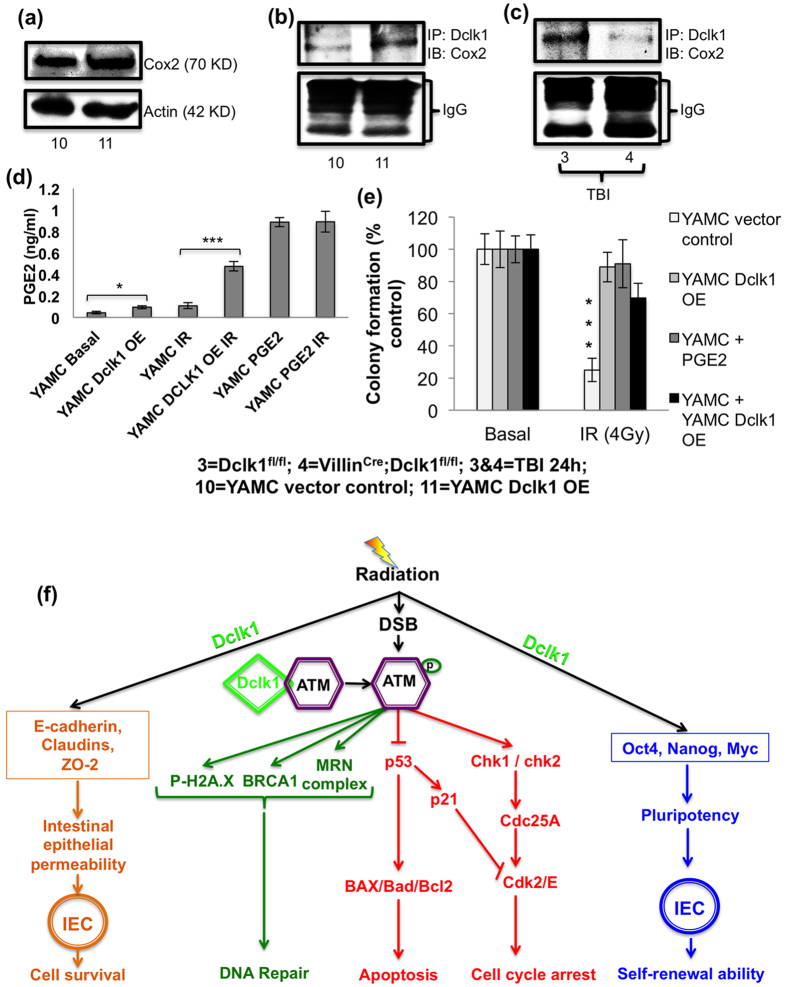
Tuft cells require Dclk1 expression to mediate its paracrine function. (**a**) Western blot analysis was performed to identify the protein expression level of COX2 in the vector control and DCLK1-overexpressing YAMC cells. (**b**) Co-immunoprecipitation: Protein extracts from vector control YAMC cells and Dclk1-overexpressing cells at basal conditions were co-immunoprecipitated with anti-Dclk1 and blotted with antibody to COX2. Lower bands represent IgG heavy and short chains. (**c**) Co-immunoprecipitation: IEC protein extracts from Dclk1^f/f^ mice at homeostasis and 24 h post-TBI were co-immunoprecipitated with anti-Dclk1 and blotted with antibody to Cox2. Lower bands represent IgG heavy and short chains. (**d**) PGE2 EIA assay: Cell culture spent media collected and utilized for analyzing PGE2 levels. (**e**) Bar graph represents the percent colony formation of YAMC cells of vector control, Dclk1-overexpressing, PGE2-treated, and co-culture (YAMC + YAMC Dclk1-overexpression) cells at baseline and 48 h post-radiation. (**f**) Proposed Mechanism: Tuft cells regulates ATM mediated DDR in response to injury to enhance intestinal epithelial survival and self-renewal for effective restitution and function. In response to radiation-induced injury, Dclk1^+^ tuft cells enhance COX2 signaling for the paracrine regulation of IECs/ISCs. Dclk1 expression is critical for ATM activation for the DNA DSB repair response. ATM activation significantly controls DNA repair, apoptosis, and the cell cycle. Cell survival and self-renewal depends upon cellular DNA integrity. Dclk1 regulation of the ATM pathway enhances cell survival and self-renewal for effective intestinal regeneration after TBI.

Finally, we sought to answer how this small population of Dclk1 expressing tuft cells regulates the whole intestinal epithelium during or after injury. From our mouse model, we learned that COX2 signaling, involved in PGE2 synthesis for paracrine regulation of IECs, was reduced in the Villin^Cre^;Dclk1^f/f^ mice compared with Dclk1^f/f^ mice 24 h and 84 h post-TBI. We observed that COX2 expression and Dclk1/COX2 interaction in Dclk1-overexpressing YAMC cells increases, whereas Dclk1/COX2 interaction decreases, in the IECs of Villin^Cre^;Dclk1^f/f^ mice compared with IECs of Dclk1^f/f^ mice 24 h post-TBI (Fig. 9a–c). We found increased levels of PGE2 in the spent medium from Dclk1-overexpressing YAMC cell culture than in that of vector control YAMC cells, both at baseline and after radiation injury (Fig. 9d). Further, we found that PGE2-treated cells showed similar colony-forming abilities to Dclk1-overexpressing YAMC cells after radiation injury (Fig. 9e). Finally, we observed that co-culture of Dclk1-overexpressing YAMC cells rescued the survival/viability of vector control YAMC cells by increasing their colony-forming abilities after radiation injury (Fig. 9e). These findings suggest that the coordinated regulation of IECs by Dclk1 expressing tuft cells during injury may occur through the paracrine action of prostaglandins. This possibility should be investigated further. Together, these data provide evidence (Fig. 9f) that (i) Dclk1-expressing tuft cells are required for coordinated regulation and/or paracrine regulation of IECs’/ISCs’ radio-resistance critical for their survival and regeneration, and (ii) Dclk1 expression regulates IECs/ISCs survival *via* enhancing DDR during/after injury.

## Discussion

ISCs are relatively resistant to radiation and other injuries due to their effective DDR, which protects their genomic integrity^7^,^26^. Dclk1 has recently been identified as a marker of tuft cells^18^ in intestinal crypts. However, the role of Dclk1 expression in tuft cell function particularly after radiation injury remains largely unknown. Our earlier report demonstrated that intestinal epithelial-specific Dclk1 deletion reduced overall animal survival 5 days post-TBI^12^. In the present study, utilizing mechanistic approaches, we discovered that loss of Dclk1 expression in the tuft cell leads to crypt hypoplasia, reduced IEC self-renewal and survival, correlated with reduced activation of the ATM mediation DDR after radiation-induced injury. We selected two critical time points to identify the importance of Dclk1 expression in tuft cells for the coordinated regulation of intestinal injury-induced repair/restitution: 24 h post-TBI, at which time p53-independent survival of ISCs is required for normal intestinal crypt recovery, and 84 h post-TBI, prior to crypt fission (96 h), which is required for efficient regeneration^9^,^23^,^45^.

We found that Dclk1 plays a pivotal role in self-renewal by protecting against DNA damage-induced chromosomal instability, permitting efficient transfer of genomic material to ISC progeny during regeneration. Furthermore, we found that as early as 24 h post-TBI, Dclk1 expressing tuft cells regulates the Bmi1^+^, Lgr5^+^, and Musashi1^+^ stem cell pools that likely cooperatively contribute to the crypt epithelial renewal process after severe injury. However, at 84 h post-TBI, only Bmi1 expression returns to baseline physiological levels, presumably to compensate for the loss of stem cell pools in Dclk1-deficient mice. Indeed, Lgr5 expression levels were decreased even before injury in Dclk1-deficient mice, suggesting that the expression of the stem cell marker Lgr5 requires Dclk1 expression in tuft cells during normal homeostasis. Consistent with these findings, the expression of putative stem cell markers Lgr5, Bmi1, and Musashi1 were reduced after siRNA-mediated knockdown of DCLK1 in colon and pancreatic cancer models^46^,^47^.

Although DCLK1 has recently been identified as having a key role in regulating pluripotency factors in cancer cells, our current findings demonstrate that Dclk1 is essential for maintaining the expression of epithelial pluripotency factors and self-renewal during crypt regeneration after injury. Although the self-renewal ability of IECs in Villin^Cre^;Dclk1^f/f^ mice is not equivalent to that found in Dclk1^f/f^ mice, the intestinal crypt architecture of the Villin^Cre^;Dclk1^f/f^ mice appears normal compared with the crypt morphology of the Dclk1^f/f^ mice under homeostasis. Therefore, Dclk1-expressing tuft cells may be dispensable during normal crypt homeostasis. However, reduced self-renewal during homeostasis may be the key mechanism for ineffective self-renewal and epithelial recovery post-TBI. Crypt hypoplasia and ineffective epithelial recovery post-TBI in Villin^Cre^;Dclk1^f/f^ mice are likely due to increased cell cycle arrest and p53-independent apoptosis. This observation seems to support the notion that apoptosis induced 24 h post-radiation is p53-independent in IECs^24^.

Under physiological conditions, pro-survival pathways, such as Wnt, Akt, mTOR, and NFκB, are involved in intestinal crypt epithelial homeostatic processes, including stem cell regulation and lineage specification^35^,^48^. However, following severe injuries, ISC function depends on the coordinated and enhanced expression of survival factors and activation of key signaling pathways that could promote cell survival, rapid proliferation, and differentiation^35^,^49^,^50^,^51^. Here, we demonstrate that loss of Dclk1 expression in tuft cells results in increased crypt apoptosis after irradiation, which was associated with significantly depressed molecular signals and impaired barrier function. After TBI, Akt/mTOR and their active forms, Notch, beta-catenin, NFkB, and their downstream targets were markedly downregulated in IECs from Villin^Cre^;Dclk1^f/f^ mice compared with IECs from Dclk1^f/f^ mice. Intestinal epithelial integrity is a necessary component in maintaining intestinal epithelial homeostasis and during regeneration after injury^52^,^53^. Loss of intestinal epithelial integrity and increased permeability due to impaired expression of tight and adherens junction proteins in Villin^Cre^;Dclk1^f/f^ mice, even 3.5 days post-TBI, may account for defective restitution responses. The maintenance of the intestinal epithelial barrier is critical for the survival of intestinal crypts; failure of the same due to loss of Dclk1 reduced survival after TBI^12^. These data are consistent with recent studies reporting the importance of Rho kinase and R-spondin in regulating the intestinal epithelial barrier after radiation injury^52^,^54^.

Here, we proposed that the impaired intestinal epithelial survival and restitution in Dclk1 knockout animals was due to a defective DDR. Deficient DDR has been suggested to increase intestinal epithelial radio-sensitivity and loss of survival^55^,^56^. ISCs are relatively radio-resistant and can more efficiently repair DNA DSBs than any other intestinal cells^5^,^9^. In early response to DNA damage, ATM and its downstream target H2AX are activated, generating gamma-H2AX and other adaptors, providing a stage for efficient HR repair^6^,^57^. Recently, ATM knockout or loss of Rad50 and Mre11 was reported to increase intestinal injury and lethality^58^,^59^,^60^. We observed a reduction in the expression of ATM, gamma-H2AX, and downstream adopter proteins BRCA1, Rad50, and MRE11 in IECs from Villin^Cre^;Dclk1^f/f^ mice 24 h post-TBI. This reduction persisted up to 3.5 days post-TBI. These data are consistent with results from recent studies reporting the importance of Bmi1 in regulating the intestinal DDR after radiation injury^61^. Therefore, profound defects in intestinal DDR in Villin^Cre^;Dclk1^f/f^ mice might contribute to defective epithelial self-renewal and increased apoptosis, which could affect intestinal epithelial regeneration in response to injury. Genomic stability is necessary for cell and organism longevity. Any defect in genomic stability, replication errors, and direct forms of DNA damage can induce mutation, which either reduces cell survival or leads to cellular transformation towards neoplasia^5^,^62^. We suggest that the expression of Dclk1 in intestinal tuft cells is critical for maintaining the genomic stability of IECs and ISCs for their survival and function, during or after injury. Thus, Dclk1 may confer radio-resistance to protect IECs and ISCs after severe genotoxic injury through regulation of the DDR.

We observed that ATM (phospho-ATM) activation is reduced in IECs from Villin^Cre^;Dclk1^f/f^ mice, under physiological conditions, and discovered that Dclk1 and ATM proteins interact in IECs from Dclk1^f/f^ mice. When compared with normal physiological homeostasis, TBI reduced the Dclk1-ATM interaction, which may be due to fewer Dclk1 expressing tuft cells remaining after TBI. However, ATM activation during and or after radiation injury directly depends on the ratio of Dclk1-ATM interaction. *In vitro* Dclk1 knockdown and overexpression experiments with YAMC cells confirmed that Dclk1 is critical for ATM activation. This study is the first to demonstrate a direct link between Dclk1 and ATM mediated DDR, which is essential for the ISC survival response to severe genotoxic injury, permitting appropriate self-renewal for effective restitution.

Dclk1 expressing tuft cells constitute a small population in the whole intestine. How this small population of Dclk1 expressing tuft cells regulate the intestinal epithelium during or after injury is unclear. Very recently, Garrett group and Locksley group uncovered an unexpected role for Dclk1 expressing tuft cells in regulating epithelial remodeling associated with type 2 immunity in the small intestine post parasite infection^63^,^64^. They further suggested that Dclk1 expressing tuft cells-derived IL-25 regulates the intestinal epithelial cell fate decision *via* a paracrine mechanism by modulating IL4 and IL13 responses derived from ILC2, an innate lymphoid cell. Jay group also supported the report with similar experimental models^21^. These studies demonstrate the importance of Dclk1 expressing tuft cells in regulating epithelial remodeling and mucosal fate after infection, even though they represent a small population in the whole intestine. Consistent with these findings, we found that this small population of Dclk1 expressing tuft cells is important in regulating intestinal epithelial self-renewal and survival by regulating DDR after radiation injury. We discovered the paracrine regulatory role of Dclk1expressing tuft cells in the small intestine. The presence of Dclk1 expression enhances COX2 signaling for prostanoids production for the paracrine regulation of IECs and ISCs in response to injury. Prostanoids are vital players regulating the epithelial injury repair response. PGE2 produced by either COX1 or COX2 is functionally radioprotective in the intestine epithelium, besides regulating DDR^43^,^65^,^66^. Together with these earlier findings, the results from the present study support the idea that Dclk1expressing tuft cells are involved in the paracrine regulation of the intestinal crypt microenvironment in response to injury, for immediate recovery response and for effective epithelial survival and regeneration.

Our findings have substantial implications for intestinal diseases, including inflammatory bowel disease (IBD), mucositis, radiation enteritis, proctitis, and colorectal cancer. Here, we suggest that Dclk1 expressing tuft cells regulate IECs and ISCs through paracrine regulation during or after injury. We suggest that Dclk1 expression may be required for an adequate DDR, thus activating the ATM pathway after injury, to protect genomic integrity. This feature is central to cell fate after significant mutagenic insult, that is, whether cell survival or death will result. If this process, however, results in survival of a mutagenic cell with self-renewal and clonal capacity, neoplastic transformation may follow. These studies implicate Dclk1 as a regulator of ISC fate, well beyond its role as a marker of tuft cells for crypt epithelial restitution. These findings may give new insight into the functional role of Dclk1 expression in tuft cells in maintaining cell and genome integrity and survival for epithelial remodeling and cell fate decision after injury.

## Materials and Methods

### Mice

All mice were age- and sex-matched and housed under controlled conditions, including a 12 h light-dark cycle, with ad libitum access to food and water. All animal experiments were performed with the approval and authorization of the Institutional Review Board and the Institutional Animal Care and Use Committee, University of Oklahoma Health Sciences Center. All the experiments were performed in accordance with relevant guidelines and regulations.

### Generation of *Villin* ^*Cre*^ *;Dclk1* ^*flox/flox*^ Mice

The *Dclk1tm1.2Jgg*/J mouse (Dclk1^flox/flox^), which possesses loxP sites flanking exon 3 of the mouse Dclk1 gene^67^, and the *B6.SJL-Tg(Vil-cre)997Gum/J* mouse (Villin^Cre^), which expresses Cre recombinase under control of the villin promoter^68^, were purchased from The Jackson Laboratory, Bar Harbor, Maine. These two mouse strains were then crossed to obtain the *Villin*^*Cre*^*;Dclk1*^*flox/flox*^ compound mouse, in which Dclk1 is specifically ablated in the intestinal epithelium. The successful Cre-recombinase-initiated DNA recombination was confirmed through genotyping of DNA from tail biopsies and intestinal tissue utilizing a PureLink^®^ Genomic DNA Isolation kit (Invitrogen, Grand Island, NY). The absence of mRNA and protein expression was confirmed by RT-PCR and western blot, respectively.

### Epithelial Crypt Isolation

As previously described^69^,^70^, small intestines were attached to a paddle and immersed in Ca2 + -free standard Krebs-buffered saline at 37 °C for 15–20 min, then gassed with 5% CO_2_, 95% O_2_. Individual crypt units were then separated by intermittent (30 sec) vibration into ice-cold phosphate-buffered saline. Then, the isolated crypts were collected by centrifugation and were processed for molecular analysis.

### Clonogenic Assay

Isolated IECs cells were plated at a density of 100 cells per well in 48-well plates in RPMI medium containing 0.3% soft agar with 1% fetal calf serum. The cell suspensions were plated in a 48-well plate above a layer of solidified 1% soft agar in plain RPMI medium. The plates were incubated at 37 °C under 5% CO_2_. The cells were followed for enterospheres formation as described previously^22^,^70^.

### RNA Isolation and Real-time RT-PCR

Total RNA isolated from small intestines was subjected to reverse transcription using Superscript™ II RNase H-Reverse Transcriptase and random hexanucleotide primers (Invitrogen). The complementary DNA (cDNA) was subsequently used to perform real-time polymerase chain reaction (PCR) by SYBR™ chemistry (Molecular Probes, Eugene, OR) for specific transcripts using gene-specific primers and JumpStart™ Taq DNA polymerase (Sigma-Aldrich, St. Louis, MO). We noted the crossing threshold value for the transcripts and normalized these with β-actin messenger RNA (mRNA). The quantitative changes in mRNA were expressed as fold-change relative to control ± *SD* value.

### Immunoprecipitation and Western Blot Analysis

For immunoprecipitation studies, IEC extracts were normalized for protein concentration and pre-cleared with 30 μl of protein A/G-coated Sepharose beads for 1 h at 4 °C. Immunoprecipitation was carried out by incubating the fractions overnight at 4 °C with antibody recognizing Dclk1. Immune complexes were captured by incubation with 50 μl of protein A/G-Sepharose beads for 2 h at 4 °C. Control experiments were performed by conducting immunoprecipitations in the presence of the control IgG antisera. The immunoprecipitated proteins were recovered by boiling the Sepharose beads in 2X SDS sample buffer.

Total IEC extracts or immunoprecipitated proteins were subjected to SDS-PAGE. Twenty-five micrograms of the total protein were size-separated in a 7.5–15% SDS polyacrylamide gel and transferred electrophoretically onto a PVDF membrane with a wet blot transfer apparatus (Bio-Rad, Hercules, CA). The membrane was blocked in 5% non-fat dry milk for 1 h and probed overnight with a primary antibody. Subsequently, the membrane was incubated with horseradish peroxidase-conjugated secondary antibody for 1 h at room temperature. The proteins were detected using ECL western blotting detection reagents (Amersham-Pharmacia, Piscataway, NJ). Actin (42-kD) was used as loading control and identified using a goat polyclonal IgG (Santa Cruz Biotechnology Inc., Dallas, Texas).

### Photo-cross-linking Label Transfer Assays

Photo-cross-linking experiments were conducted per the manufacturer’s protocol (Label Transfer Kit, Thermo Scientific, IL, USA). Recombinant DCLK1 was incubated with Sulfo-SBED [sulfosuccinimidyl [2–6-(biotinamido)-2-(p-azidobenzamido)-hexanoamido] ethyl-1,3′-dithiopropionate] in dimethyl fluoride to conjugate the cross-linking reagent. The excess cross-linking reagent was removed by dialyzing the reaction mixture against the label transfer buffer. SBED-labeled-DCLK1 was mixed with recombinant ATM protein and incubated for 30 min at room temperature. The reaction mixture was exposed with a UV light for 10 min. Samples were equally split and an appropriate amount of 4X SDS sample buffer (lacking DTT and β-mercaptoethanol) was added. The reaction mixtures were incubated for 2 min at room temperature. In one set of samples, DTT was added at a final concentration of 100 mM. Samples were mixed and heated in the tubes for 5 min. Each reaction mixture was loaded on a 4–20% gradient polyacrylamide gel. The proteins were transferred to polyvinylidene difluoride (PVDF) membrane, blocked for 30 min, probed with ATM antibody for 3 h, and subsequently probed with respective secondary antibody for 1 h. After the membrane was rinsed with PBS-T, the blot was developed using enhanced chemiluminescence. Following detection, the membrane was stripped and probed with a 1:5,000 dilution of streptavidin-HRP for 1 h. After the proteins were rinsed with PBS-T, the blot was developed by enhanced chemiluminescence.

### Lentiviral Constructs and Lentivirus Production

As previously described^46^, Dclk1 isoform long-α cDNA tagged with GFP and GFP cDNA was amplified and ligated into pCR8-GW-TopoD (Invitrogen), following the manufacturer’s protocol. Wild-type Dclk1-GFP and GFP cDNAs were then transferred to pLenti CMV PURO DEST empty (gift from Dr. Eric Campeau) using Clonase 2 (Invitrogen), following the manufacturer’s recommendations. The expression plasmids constructed above were co-transfected along with packaging plasmids pMD2.G (Addgene), pMDL/RRE g/p (Addgene), and pRSV-Rev (Addgene) into 293 T cells. DNA was transfected into cells using the PEI transfection method, with a PEI to DNA ratio of 1:1. Supernatants were harvested 48 and 72 h post-transfection and cleared through a 0.45-μm filter. These viral supernatants were concentrated using polyethylene glycol 8000 (Sigma-Aldrich). Cells were infected with concentrated virus and selected with puromycin (Sigma-Aldrich) to establish stable cell lines.

### Colony Formation Assay

Dclk1**-**overexpressing YAMC cells (5000/well) or control vector cells were seeded into 6-well plates in cell culture medium containing 10% FBS. After 24 h, cells were exposed to IR (4 Gy). The cells were allowed to grow for 24 h, washed with PBS, and passaged into new 6-well plates (100 cells/well). Cells were allowed to grow for one week, then were fixed with glacial acetic acid/methanol solution (1:3) and washed with PBS. Colonies were stained with 0.5% crystal violet for 10 min and washed with tap water to remove excess stain. Colonies were then counted under a stereomicroscope using a 1 cm^2^ grid. Four squares from four quadrants were counted for each well.

### DNA Fragmentation Assay

IECs or YAMC cells were collected by centrifugation at different time points after irradiation. Genomic DNA samples were isolated using the PureLink Genomic DNA kit (Life Technologies, CA, USA). Cells were resuspended in binding buffer and ethanol, and were rinsed prior to the elution of purified DNA. For each sample, the same amount of DNA (200 ng) was loaded on a 1.5% agarose gel containing 0.5 μg/ml ethidium bromide. Electrophoresis was performed. The DNA in the gels was visualized under ultraviolet light and photographed using an Alpha Innotech Gel Imager.

### Immunohistochemistry

Heat-induced epitope retrieval was performed on 4-μm formalin-fixed, paraffin-embedded sections utilizing a pressurized Decloaking Chamber (Biocare Medical LLC, Concord, CA) in citrate buffer (pH 6.0) at 99 °C for 18 minutes.

### Brightfield

Slides were incubated in 3% hydrogen peroxide at room temperature for 10 min. After incubation with primary antibody overnight at 4 °C, slides were incubated in a Promark peroxidase-conjugated polymer detection system (Biocare Medical LLC) for 30 min at room temperature. After washing, slides were devolved with diaminobenzidine (Sigma–Aldrich).

### Fluorescence

Slides were incubated in a normal serum and BSA blocking step at room temperature for 20 min. After incubation with primary antibody overnight at 4 °C, slides were labeled with Alexa Fluor^®^ dye-conjugated secondary antibody and mounted with ProLong Gold (Invitrogen).

### Image Acquisition

Slides were examined on the Nikon Eclipse Ti motorized microscope paired with the DS-Fi2 color and CoolSnap ES2 monochrome digital cameras utilizing DIC-enhanced PlanApo objectives operated by the NIS-Elements Microscope Imaging Software platform (Nikon Instruments, Melville, NY).

### PGE2 Measurement

The PGE2 levels in the serum and spent cell culture media were measured using a PGE2 EIA kit (Cayman Chemical Company, Ann Arbor, MI, USA). The concentration of PGE2 was expressed as ng/ml. Each sample was assayed in triplicate.

### Antibodies

The following antibodies were used: Dclk1, BrdU, Lgr5, Bmi1, Msi1, COX1, COX2, Claudin1, Claudin5, Claudin7 [Abcam, Cambridge, MA]; Claudin-7, E-Cadherin, β-actin, Notch1, NFkB, Betacatenin, [Cell Signaling, Danvers, MA]; Alexa Fluor^®^ 488 donkey anti-rabbit IgG (A-21206), Alexa Fluor^®^ 568 Donkey Anti-Goat IgG (A-11057), Hoechst 33342 DNA stain (H1399) [Invitrogen].

### Statistical Analysis

All the experiments were performed in accordance with relevant guidelines and regulations. All experiments were performed in triplicate. Results were reported as average + /− *SEM*. Data was analyzed using the Student’s *t*-test for comparison of mean values between groups. *P* values of <0.05 = *, <0.01 = **, and 0.001 = *** were considered statistically significant. For multiple comparisons, one-way ANOVA followed by Newman-Keuls test was performed.

## Additional Information

**How to cite this article**: Chandrakesan, P. *et al*. Intestinal tuft cells regulate the ATM mediated DNA Damage response via Dclk1 dependent mechanism for crypt restitution following radiation injury. *Sci. Rep.* **6**, 37667; doi: 10.1038/srep37667 (2016).

**Publisher’s note:** Springer Nature remains neutral with regard to jurisdictional claims in published maps and institutional affiliations.

## Supplementary Material

Supplementary Figures
