# Supplementary material for: Intestinal tuft cells regulate the ATM mediated DNA Damage response via Dclk1 dependent mechanism for crypt restitution following radiation injury
Source: Sci Rep. 2016 Nov 23;6:37667. doi: 10.1038/srep37667 (PMC5120335; doi:10.1038/srep37667)
Supplement: Supplementary Figures [file srep37667-s1.doc]

**Intestinal tuft cells regulate the ATM mediated DNA Damage response via Dclk1 dependent mechanism for crypt restitution following radiation injury**

Parthasarathy Chandrakesan^1,2,3^, Randal May^1,3^, Nathaniel Weygant^1^, Dongfeng Qu^1,2^, William L. Berry^2^, Sripathi M. Sureban^1,3^, Naushad Ali^1,3^, Chinthalapally Rao^1,2^, Mark Huycke^1,3^, Michael S. Bronze^1^ and Courtney W. Houchen^1,2,3,4^

^1^Department of Medicine, University of Oklahoma Health Sciences Center, Oklahoma City, OK 73104, USA; ^2^OU Cancer Institute, University of Oklahoma Health Sciences Center, Oklahoma City, OK 73104, USA; ^3^Department of Veterans Affairs Medical Center, Oklahoma City, OK 73104, USA; ^4^COARE Biotechnology, Inc., Oklahoma City, OK 73104, USA.

**Supplementary Figure 1:**

**
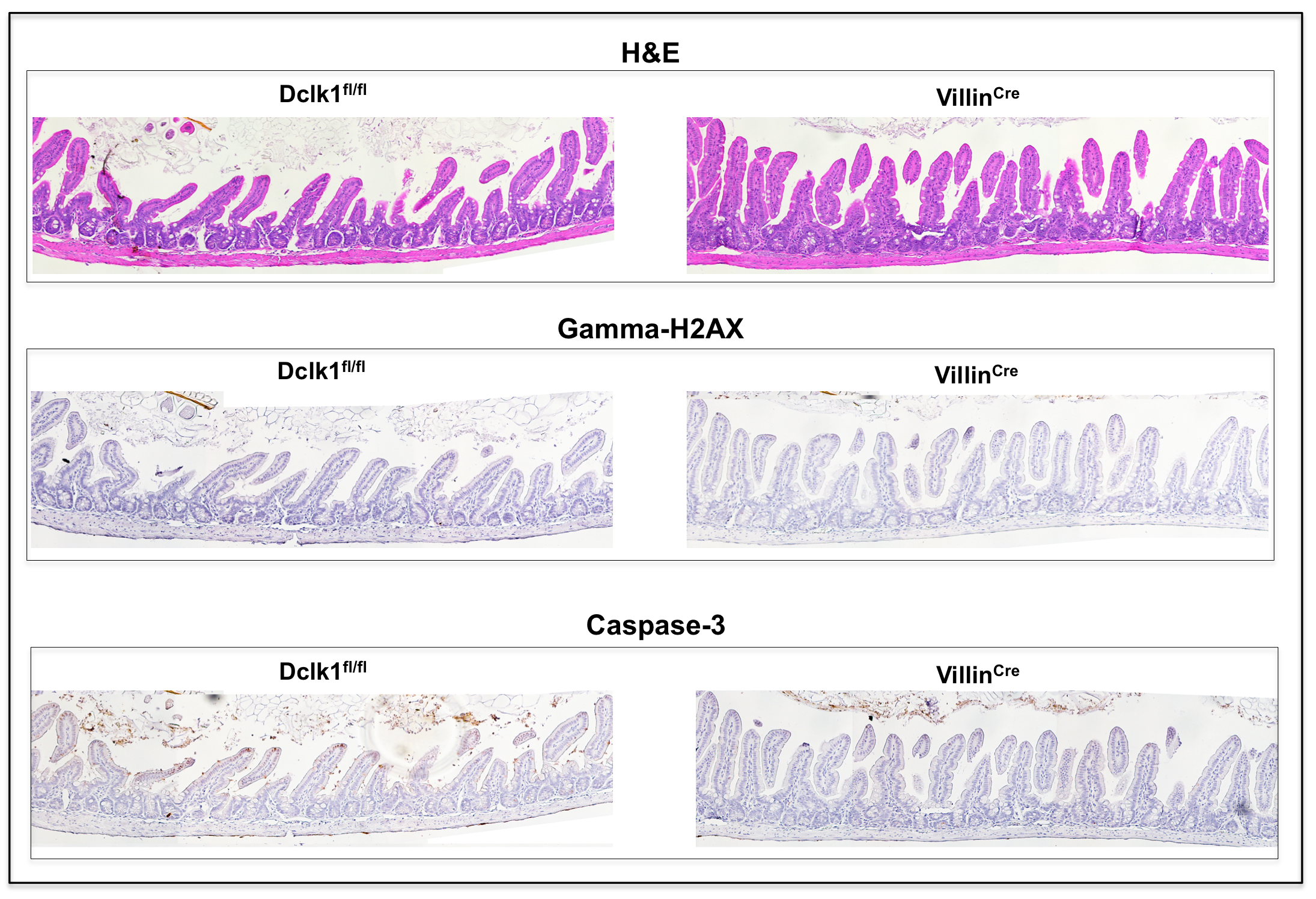
**

**Supplementary Figure 1. No change in the intestinal epithelial phenotype and DNA damage between Villin^Cre^ mice and Dclk1^f/f^ mice before radiation injury.** Intestinal tissue sections from Villin^Cre^ mice and Dclk1^f/f^ mice, were stained for H&E. To assess DNA damage/apoptosis, intestinal tissue sections from Villin^Cre^ mice and Dclk1^f/f^ mice, were stained for Gamma-H2AX and Cas3.

**Supplementary Figure 2:**


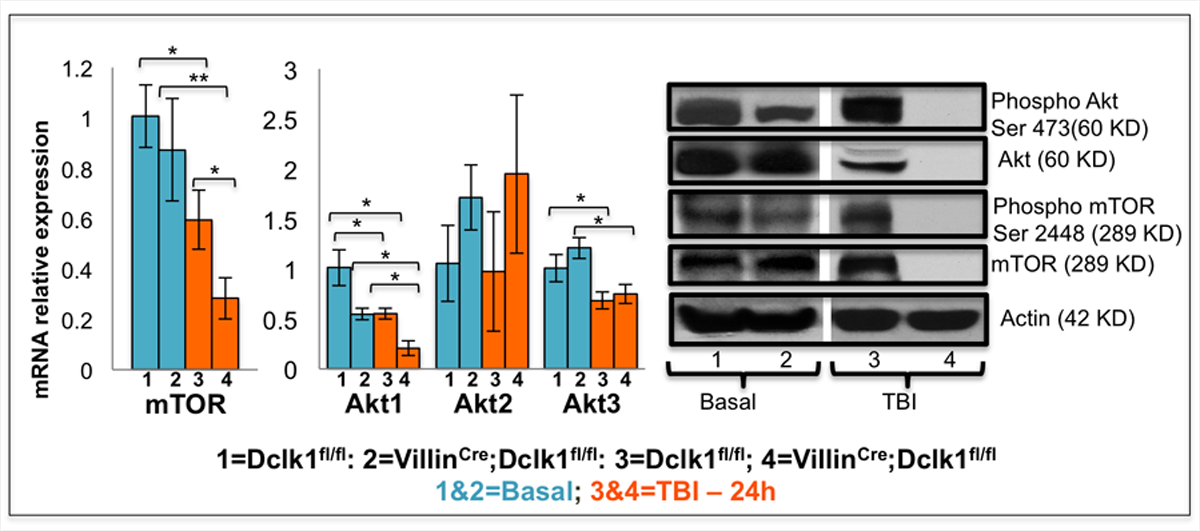


**Supplementary Figure 2. Intestinal epithelial survival signaling pathways of Akt/mTOR require Dclk1 expressing tuft cells for injury response.** RT-PCR analysis of mRNA expression of mTOR/Akt (Akt1/Akt2/Akt3) in IECs isolated from Villin^Cre^;Dclk1^f/f^ and Dclk1^f/f^ mice, before and 24 h after TBI. Western blot analysis of protein expression of phospho Akt (ser 473), total Akt, phospho mTOR (ser 2448), and total mTOR in IECs isolated from Villin^Cre^;Dclk1^f/f^ and Dclk1^f/f^ mice, before and 24 h after TBI. All quantitative data are expressed as means ± *SD* of a minimum of three independent experiments. *P* values of <0.05 = *, <0.01 = **, and 0.001 = *** were considered statistically significant.

**Supplementary Figure 3:**


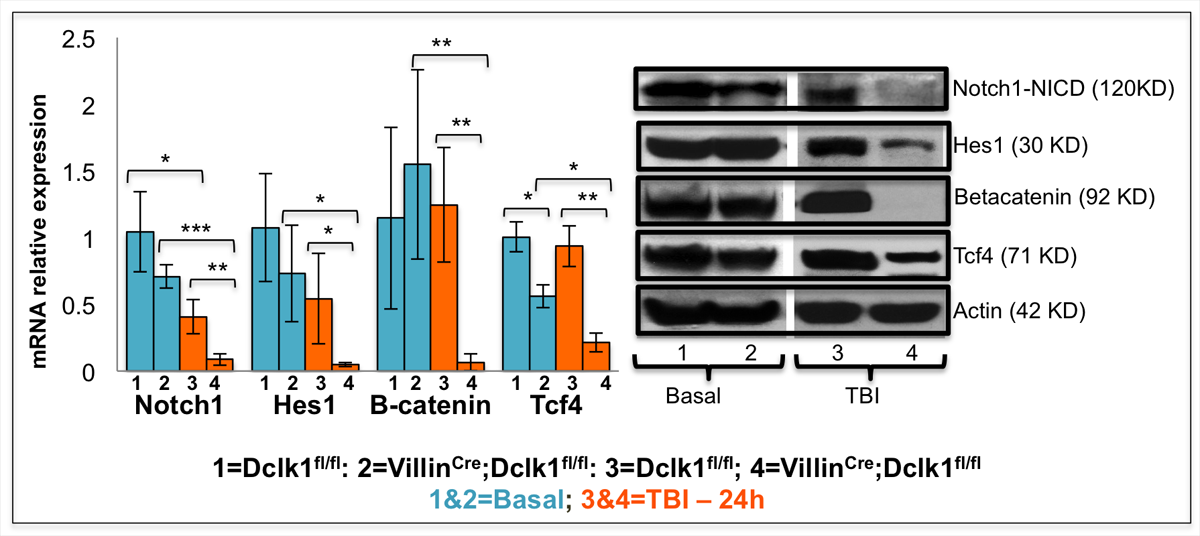


**Supplementary Figure 3. Intestinal epithelial survival signaling pathways of Notch/Betacatenin require Dclk1 expressing tuft cells for injury response.** mRNA expression by RT-PCR analysis of Notch1, Hes1, B-catenin, and Tcf4 in IECs isolated from Villin^Cre^;Dclk1^f/f^ and Dclk1^f/f^ mice, before and 24 h after TBI. Western blot analysis of protein expression of Notch1 (NICD), Hes1, B-catenin, and Tcf4 in IECs isolated from Villin^Cre^;Dclk1^f/f^ and Dclk1^f/f^ mice, before and after 24 h TBI. All quantitative data are expressed as means ± *SD* of a minimum of three independent experiments. *P* values of <0.05 = *, <0.01 = **, and 0.001 = *** were considered statistically significant.

**Supplementary Figure 4:**

**
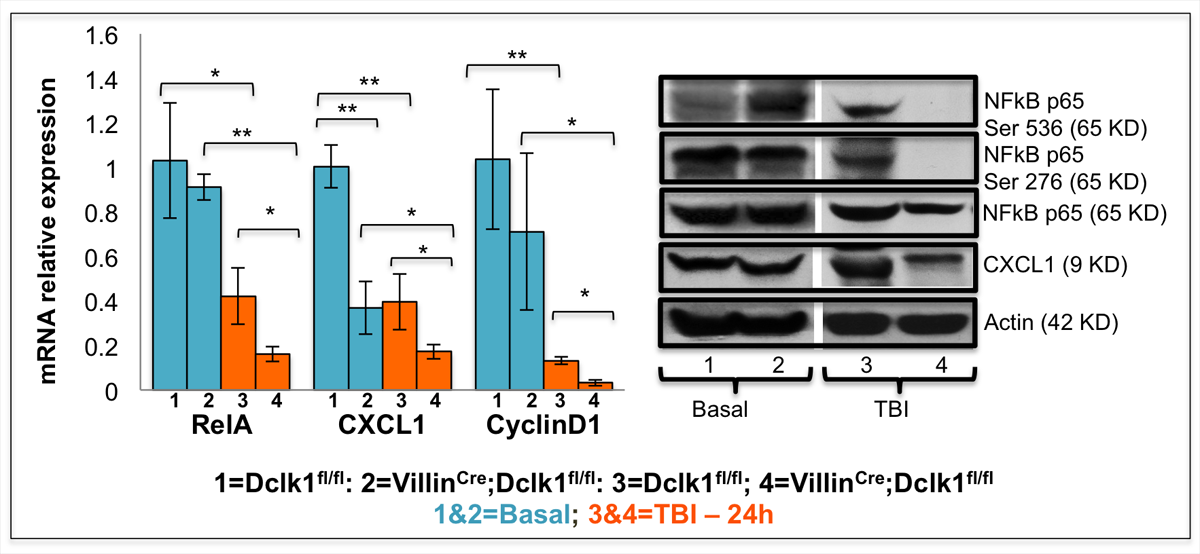
**

**Supplementary Figure 4. Intestinal epithelial survival signaling pathway of NFkB require Dclk1 expressing tuft cells for injury response.** RT-PCR analysis of mRNA expression of NFkB pathway RelA (NFkBp65), CXCl1, and CyclinD1 in IECs isolated from Villin^Cre^;Dclk1^f/f^ and Dclk1^f/f^ mice, before and 24 h after TBI. Western blot analysis of protein expression of phospho NfkBp65 (ser 536 and ser 276), total Nfkbp65, and CXCl1 in IECs isolated from Villin^Cre^;Dclk1^f/f^ and Dclk1^f/f^ mice, before and after 24 h TBI. All quantitative data are expressed as means ± *SD* of a minimum of three independent experiments. *P* values of <0.05 = *, <0.01 = **, and 0.001 = *** were considered statistically significant.

**Supplementary Figure 5:**

**
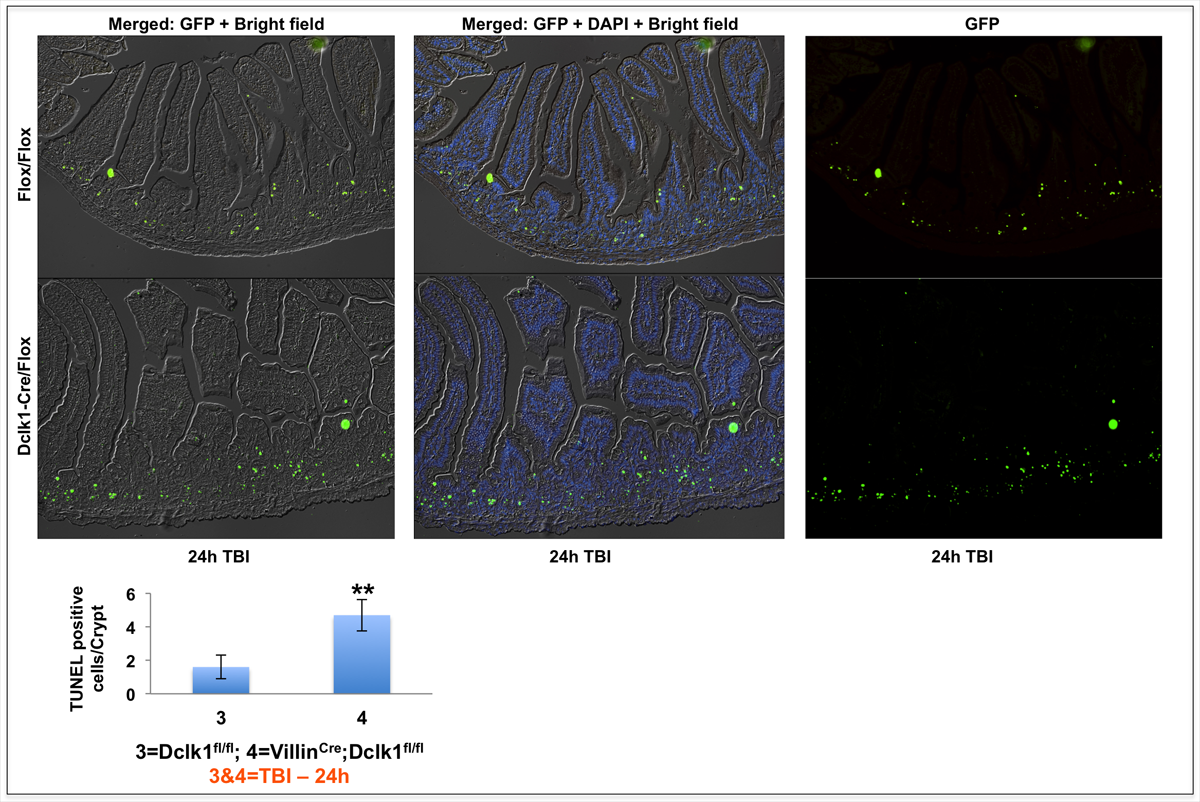
**

**Supplementary Figure 5. Dclk1 deficiency increased intestinal epithelial apoptosis 24h post TBI.** To assess apoptosis, intestinal tissue sections from Villin^Cre^;Dclk1^f/f^ mice and Dclk1^f/f^ mice, before and 24 h after TBI, were TUNEL-stained. Bar graph represents the score of TUNEL positive cells/crypt. All quantitative data are expressed as means ± *SD* of a minimum of three independent experiments. *P* values of <0.05 = *, <0.01 = **, and 0.001 = *** were considered statistically significant.

**Supplementary Figure 6:**

**
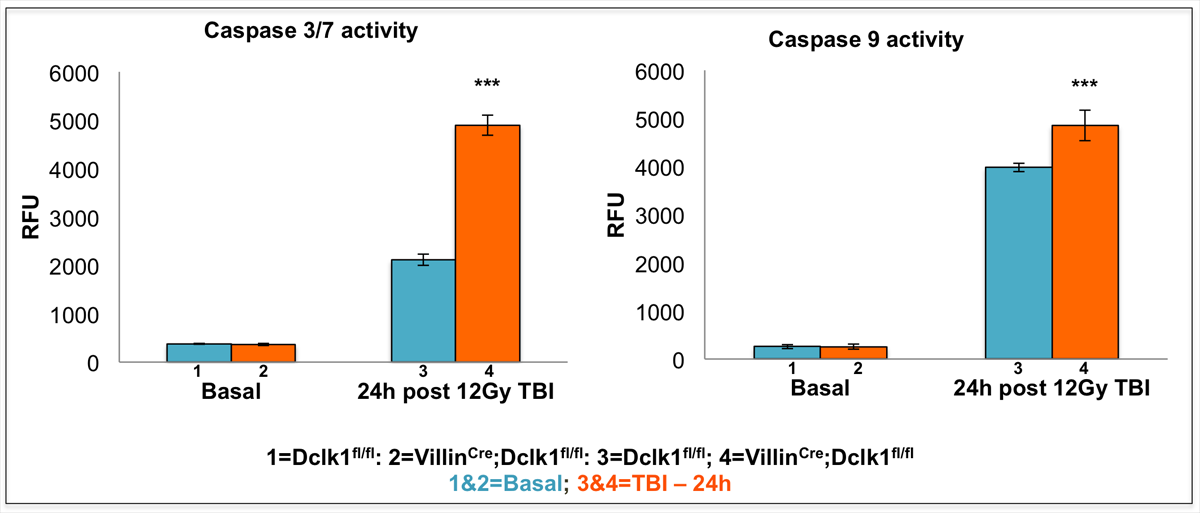
**

**Supplementary Figure 6. Dclk1 deficiency increased intestinal epithelial apoptosis 24h post TBI.** To assess IEC apoptosis, Caspase 3/7 and Caspase 9 activity assay (Luminescent Caspase Glo assay kit, Promega) was performed to identify the level of caspase activities in IECs isolated from Villin^Cre^;Dclk1^f/f^ and Dclk1^f/f^ mice, before and 24 h after TBI. All quantitative data are expressed as means ± *SD* of a minimum of three independent experiments. *P* values of <0.05 = *, <0.01 = **, and 0.001 = *** were considered statistically significant.

**Supplementary Figure 7:**

**
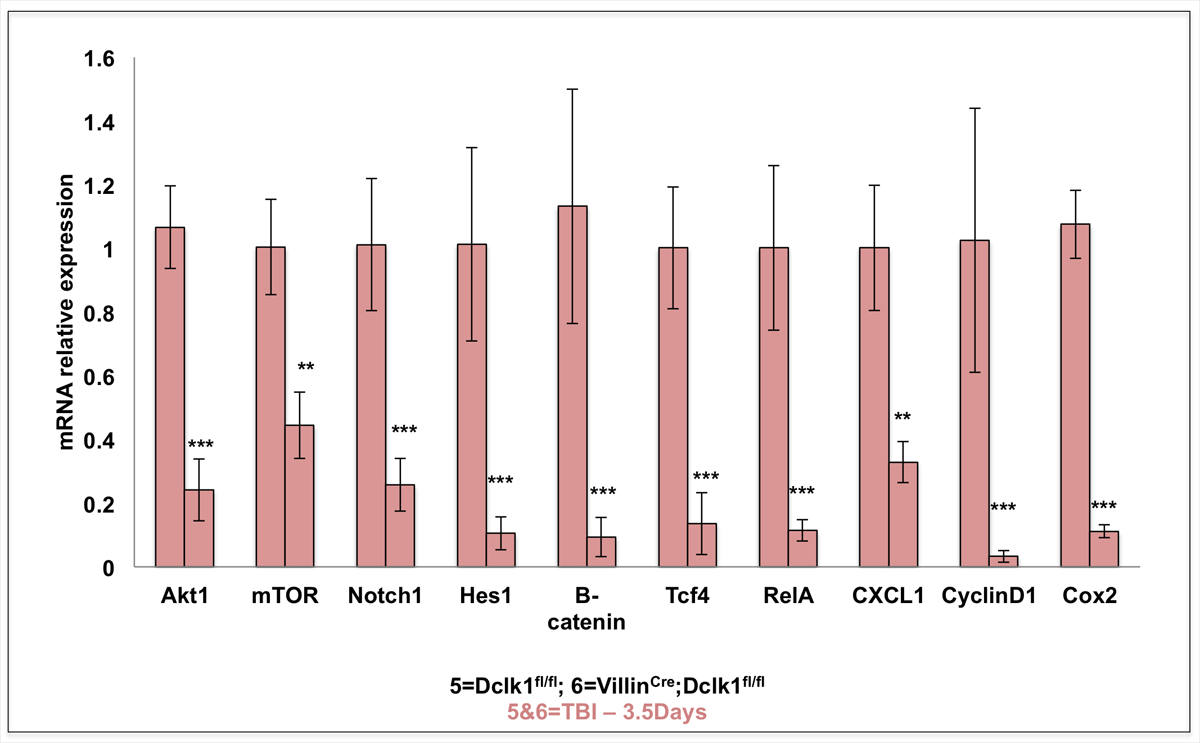
**

**Supplementary Figure 7. Dclk1 expression in tuft cells is required for its function for the enhancement of intestinal crypt epithelial cell survival factors and activation of functional metabolic signaling pathways 3.5 days post TBI.** RT-PCR analysis of mRNA expression of mTOR/Akt, Notch1, Hes1, B-catenin, and Tcf4 and NFkB pathway RelA (NFkBp65), CXCl1, CyclinD1, and Cox2 in IECs isolated from Villin^Cre^;Dclk1^f/f^ and Dclk1^f/f^ mice, 3.5 days after TBI. All quantitative data are expressed as means ± *SD* of a minimum of three independent experiments. *P* values of <0.05 = *, <0.01 = ** and 0.001 = *** were considered statistically significant.
